# Non-Small Cell Lung Cancer—Tumor Biology

**DOI:** 10.3390/cancers16040716

**Published:** 2024-02-08

**Authors:** Mumtaz V. Rojiani, Amyn M. Rojiani

**Affiliations:** 1 Department of Pathology, Penn State College of Medicine, Hershey, PA 17033, USA; arojiani@pennstatehealth.psu.edu; 2 Penn State Cancer Institute, Penn State College of Medicine, Hershey, PA 17033, USA; 3 Department of Pharmacology, Penn State College of Medicine, Hershey, PA 17033, USA

Lung cancer is one of the leading causes of cancer-related mortality worldwide among men and women [1]. It is divided mainly into two subtypes: small lung cancer (SCLC) and non-small cell lung cancer (NSCLC). NSCLC accounts for the majority (around 85%) of all lung cancer cases and includes two major types, lung adenocarcinoma (LUAD) and lung squamous cell carcinoma (LUSC), which form 70% and 30% of all the total cases, respectively [2]. The challenges of this devastating disease are manifold and range from metastatic and recurrent aggressive disease, to poor response to therapeutic options, and although chemotherapy remains a valuable adjunct [3,4], it is also impacted by recurrences that are chemoresistant and more aggressive [5]. Despite recent advances in surgery, chemotherapy, radiotherapy, and immunotherapy, the 5-year survival rate of lung cancer patients remains dismally poor [6]. Significant investigative efforts have continued to expand our understanding of the underlying biology, yet it remains elusive.

This Special Issue of Cancers has sought to compile a series of original research articles and timely, comprehensive reviews encompassing all aspects of lung cancer biology. Investigations into the myriad changes within the tumor microenvironment, including angiogenesis, invasion, epithelial–mesenchymal transition, cancer stem cells, deciphering molecular changes, and identifying biomarkers and predictors of prognosis and behavior, as well as advances in therapeutic options and resistance mechanisms, were topics of special interest, and contributions on other significant topics that further enhance our understanding of the biology of lung cancer were also invited for submission. 

The Special Issue generated significant interest and was closed with fourteen original articles and four reviews. The titles reflect the wide range of topics considered under the aegis of tumor biology, many with significant overlap, including broad categories such as the tumor microenvironment, immunogenomics, angiogenesis, therapeutics, chemoresistance, metabolic reprogramming and other factors impacting survival.

Cancer models that accurately define the biology of human tumors have been developed and modified over many decades. NSCLC-patient derived xenograft (PDX) models have added much to our understanding of the biology of these tumors and have perhaps been most extensively used to evaluate chemotherapy and targeted therapeutic assays amongst various other applications [7]. The study by Pardo-Sanchez et al. (Contribution 1) that reports an increased growth rate and mesenchymal properties of PDX during serial transplantation highlights the need for constant vigilance in recognizing changes in the biology of these models. The finding that adenocarcinoma (mostly KRAS-G12C mutated) was more likely to engraft versus other types of NSCLC, and that these models facilitate a better understanding of EMT processes, is intriguing.

Mammalian SWI/SNF (SWitch/Sucrose Non-Fermentable) complexes are ATP-dependent chromatin remodelers whose subunits have emerged among the most frequently mutated genes in cancer. In the second article, Peinado P et al. (Contribution 2) combined genomic, transcriptomic, and proteomic approaches to study the mutational status and expression levels of the SWI/SNF subunits in lung adenocarcinoma (LUAD) cell lines. They reported that the SWI/SNF complex was mutated in more than 76% of their LUAD cell lines and there was a high variability in the expression of the different SWI/SNF subunits. These findings are presented to assist investigators in selecting appropriate models for the study of SWI/SNF as a tumor suppressor in LUAD.

Liberini V et al (Contribution 3) explored NSCLC biomarkers predicting response to immunotherapy with checkpoint inhibitors (ICI). This review paper describes some of the current research on predictive factors derived from both in vitro/ex vivo analysis and in vivo analysis; ranging from conventional pathology to molecular biology, application of newest techniques in molecular imaging, exponential increase of knowledge due to technological advancements and to new bioinformatics approaches applied to image analyses.

Factors that impact survival and prognosis remain active topics for investigations and continue to be updated [8]. In this Special Issue, multiple studies have investigated factors that modulated or predicted survival in NSCLC patients. One such report by Lindenmann J et al. (Contribution 4) described the impact of preoperative oxygen consumption on survival following resection. A prognostic factor in various cardio-respiratory diseases, namely peak oxygen consumption (VO_2_ peak), was retrospectively studied in 342 patients with curatively resected NSCLC. They reported an association between a low preoperative VO_2_ peak, and both decreased postoperative overall survival and decreased non-tumor-related survival during the 10-year follow-up. Similarly, Souza CP et al. (Contribution 5) described an association of deregulated microRNAs with patient survival. The investigators used global miRNA expression profiling analysis in paired tumor and normal lung tissues. In total, 33 significantly deregulated mRNAs were reported in tumors compared with normal lung tissue. They confirmed that these 33 miRNAs targeted genes are aberrantly expressed in NSCLC. They particularly described a significant association of miR-25-3p with poor patient survival and therefore its potential as a prognostic biomarker. In this Special Issue, Goto T et al. (Contribution 6) reported the association of mutation profiles with postoperative survival in NSCLC patients. This study sought to analyze mutations in lung cancer, based on next-generation sequencing data of surgically resected tumors. The *TP53* allele fraction tended to be high in tumors that were topographically located in caudal and dorsal parts of the lung. *TP53*-mutated lung cancers located in segments 9 and 10 were associated with significantly poorer prognosis than those located in segments 1–8. The investigators demonstrated for the first time, that there is an association between the TP53 mutation profile and the location of the tumor and that this is also similarly associated with the postoperative prognosis. Ahluwalia P et al. (Contribution 7) describe an immunogenic gene signature of cell death-associated genes that have prognostic implications in NSCLC. In this data-mining analysis using TCGA data, the expression of genes involved in cell death pathways and resulting infiltration of immune cells was explored in lung adenocarcinoma. Genes involved in autophagy, apoptosis and necrosis were analyzed and a 21 gene cell death index (CDI) was developed, which stratified patients into high risk, high CDI and low risk, low CDI, and correlated mortality. Additional analysis highlighted the presence of an immunocompromised microenvironment indicated by the higher infiltration of cytotoxic T cells along with the presence of checkpoint molecules and T cell exhaustion genes. Patients at higher risk might be more suitable to benefit from PD-L1 blockade or other checkpoint blockade immunotherapies.

Understanding the mechanisms leading to the development of chemoresistance [9], as well as the possible alternatives and biomarkers, continues to garner the attention of many investigators. A number of investigators address these topics. Padmanabhan J et al. (Contribution 8) targeted cyclin dependent kinase 9 (CDKN9), using three different CDK9 inhibitors in the NSCLC cell line and lung cancer organoids. These compounds reduced cell survival and tumorigenesis of KRAS and EGFR mutated cell lines at very low concentrations. These CDK9 inhibitors remained effective against Osimertinib-resistant PC9 and AMG510-resistant H23 and H358 cells. Collectively, they suggest that CDK9 inhibitors would be effective against NSCLCs as well as those tumors that develop resistance to targeted therapies. Pemetrexed, an antifolate second-line chemotherapy drug, is used in the treatment of advanced NSCLC patients. In their investigation of pemetrexed-resistant NSCLC cells derived from A549 cells, the development of resistance was correlated with the expression of cancer stem-cell-related proteins such as BMI1 or CD44, along with an elevated EMT signature (Contribution 9). BMI1 inhibition by a small molecule inhibitor PTC-209 or transduction of BMI1-specific shRNAs suppressed cell growth. Along with other data, it was shown that BMI1 expression mediates pemetrexed sensitivity of NSCLC cells and thus remains a viable therapeutic consideration. In the context of chemoresistance, Pernia O et al. (Contribution 10) reported a novel role for the tumor suppressor gene *ITF2* in tumorigenesis and chemotherapy response. They identified that as cells were exposed over a long period to platinum compounds, there was frequent deletion of the *ITF2* gene. Expression of this gene re-sensitized tumor cells to platinum and recovered the levels of Wnt/β-catenin transcriptional activity, thus implying that ITF2 served as a molecular mechanism driving cells to develop chemoresistance to cisplatin, likely through the Wnt-signaling pathway. They also identified an inverse correlation between *ITF2* and *HOXD9* expression, revealing that NSCLC patients with lower expression of *HOXD9* had a better overall survival rate.

In their detailed review of intratumoral cellular heterogeneity, Gregorc V et al. (Contribution 11) discussed its implications for drug resistance in NSCLC patients. As is well known, the development of resistance to first- and second-line therapies as well as targeted treatments remains a very significant problem in managing NSCLC patients. The significant advances in technology and biologic methods such as single-cell sequencing have led to more accurate profiling of tumors. This review highlighted current research on the biological role of tumor heterogeneity and its impact on the development of acquired resistance in NSCLC patients.

In its broad definition, the tumor microenvironment’s impact on the biology of these neoplasms has been examined for many years [10]. In their comprehensive review of the renin–angiotensin system (RAS) in lung tumors and its microenvironment interactions, Catarata MJ et al. (Contribution 12) described the mechanistic involvement of this system beyond its typically perceived role in cardiovascular pathophysiology. Exploring its pleiotropic role, the review discusses the interaction of angiotensin peptides within the tumor microenvironment, impacting essential cancer biology processes. The RAS has been associated with at least six acquired, functional capabilities: sustained angiogenesis, evasion of apoptosis, self-sufficiency in growth signals, insensitivity to anti-growth signals, tissue invasion and metastasis, and limitless replication potential [11], although most prominent are its effects on angiogenesis, invasion, pro-survival signaling and proliferation. The complex interactions of the RAS that define its wide interactions is also its Achilles heel as its interactions both in the tumor microenvironment and with other chemotherapeutic interventions may be quite variable. Nonetheless, the system provides insights into additional viable targets for consideration.

Other tumor microenvironment interactions are described in the submission by Liotti F et al. (Contribution 13) in which they describe the resolution of inflammation and inhibition of angiogenesis, mediated by Toll-like receptor 7 in NSCLC. In their submission, Tubin s et al. (Contribution 14) reviewed the interplay between cancer cells, radiation therapy (RT), and the tumor immune microenvironment. This intriguing review article examines the impact of conventional radiotherapy in destroying many of the lymphocytes in the irradiated tumor environment, contributing to immune suppression, even while being cytotoxic to the tumor cells. The authors discussed the natural immunosuppressive effects of NSCLC cells and conventional RT approaches, and then shift their focus to immunomodulation through novel, emerging immuno- and RT approaches that promise to generate immunostimulatory effects to enhance tumor control and patient outcomes.

Metabolic reprogramming in cancer cells, or the Warburg effect, is a well-established hallmark in continued tumor growth and has been examined in the context of NSCLC [12]. In their study, Dyrstad SE et al. (Contribution 15) describe the effects of blocking aerobic glycolysis by targeting pyruvate dehydrogenase kinase in combination with EGFR TKI and ionizing radiation increases to address resistance to targeted EGFR tyrosine kinase inhibitors (TKI). Following transcriptome analysis, increased expression of pyruvate dehydrogenase kinase 1 (PDHK1) as well as upregulated expression of the genes involved in glucose metabolism were identified. This study sought to determine if targeting PDHK would prevent the development of resistance to EGFR TKIs in NSCLC cells and indeed the PDHK1 inhibitor dichloroacetate (DCA) in combination with EGFR TKIs and/or ionizing radiation was shown to increase the therapeutic effect in their NSCLC cell models. As the authors state, this mechanism was associated with redirected metabolism towards pyruvate oxidation and reduced lactate production, both in EGFR-TKI-sensitive and resistant NSCLC cells.

La Ferlita A et al. (Contribution 16) reported a transcriptomic analysis of human endogenous retroviruses (HERVs) in NSCLC. This class of transposable elements has been implicated in cancer development and reported as novel biomarkers for several tumor types. This study characterized the expression of HERVs at genomic locus-specific resolution in lung cancer using the TCGA. The study provides further insight into the dysregulation involving HERVs that occurs in lung cancer and may provide additional novel biomarkers. Tamirat MZ et al. (Contribution 17) describe a structural basis for the functional changes by EGFR Exon 20 insertion mutations. It is well known that activating somatic mutations of the epidermal growth factor receptor (EGFR) are implicated in NSCLC. Less commonly, exon 20 insertions V769insASV and D770insNPG are also observed in NSCLC. In the active state, the mutations increase interactions that stabilize the αC helix that is essential for EGFR activity and the insertions disrupt an interaction essential in stabilizing the inactive conformation, which could drive the kinase from an inactive to an active EGFR state.

Finally, attesting to the wide repertoire of articles received in the context of this Special Issue is an understanding of the role of lung microbiota in lung cancer [13]. In the study by Wong LM et al. (Contribution 18), a comparative analysis of the microbiome in LUAD and LUSC was carried out. The authors report an analysis of the intra-tumor microbiota, seeking differences in its composition by age and gender. Dysregulated microbes were correlated with patient survival rates, immune infiltration, immune and cancer pathways, and genomic alterations. LUAD-associated *Escherichia coli* str. K-12 substr. W3110 was dysregulated in older female and male patients and correlated with both patient survival and genomic alterations. For LUSC, the most prominent bacterial species was *Pseudomonas putida* str. KT2440, uniquely associated with young LUSC male patients and immune infiltration.

This Special Issue of Cancers titled “NSCLC—Understanding Tumor Biology” thus presents a widely varied set of research and review papers, reflecting some areas of accomplishment, while also defining areas of the biology of these tumors with which we continue to grapple. Although broad areas of common interest such as tumorigenesis, chemoresistance, biomarkers form the major areas of investigation, it is heartening to review manuscripts in less studied areas such as the intra-tumoral microbiome and metabolic reprogramming, as well as studies that tap into the vast amounts of information generated by data sets such as the TCGA and others. We remain optimistic that investigations such as the ones included in this Special Issue will continue to contribute positively to our understanding of lung cancer.
